# On Ossifying Fungous Growths or Osteoid Tumours

**Published:** 1845-04

**Authors:** 


### Art. XIV.

*Ueber ossificirende Schwämme oder Osteoid-geschwulste.* Von Joh. Müller: *gelesen in der Hufelandschen Med.-chirurg. Gesellschaft am* 1 *Sept.* 1843. *Müller's Archiv für Physiologie,* Heft V, 1843.

*On Ossifying Fungous Growths or Osteoid Tumours.* By J. Müller. *Read at the Hufeland Medico-chirurgical Society on the* 1*st of Sept.* 1843. *In Müller's Archives for Physiology, &c.* Part V, 1843. 8vo, pp. 56.

We have thought it best to treat this paper as a book, not only because of its interest and importance, but because it is too long to be put in the company of "extracts from journals," and forms a kind of appendix to the learned author's work on Cancer and other Morbid Growths, which was reviewed in our Tenth Volume. In that work he made passing mention of the "osteoid tumour of the bones which is a growth composed entirely of osseous substance," [*at page* 236]; but knowing little about it he prudently said less. The object of his present paper is to give a further account of this kind of tumours, which he has diligently continued to investigate ever since the publication of his work; and though his task is not completely done, yet it is well done, and he has supplied far more information upon this subject than could possibly be collected from all that has hitherto been published.

"The name of osteoid," he says, "is limited to designate the malignant tumours, leading to the ruin of the organism, which consist for the most part of osseous substance, and occur secondarily, not in the bones alone, but in the soft parts . . . . . . The term is well suited, because it indicates that these formations do not consist of osseous substance alone, but that there is in them another part unossified but capable of ossification." (p. 400.)

To render his description more intelligible, Müller has given reference to several plates of what he regards as osteoid tumours published under various names in former works; and, as our readers may be able to turn to some of these, we have copied the references in a note,* and add, that among the many specimens of the disease in the Hunterian Museum there is an admirable series taken from the patient of whose "ossified lung" Dr. Baillie has given a plate in his Illustrations of Morbid Anatomy. (Fasc. ii. Pl. vi, fig. 2.)

The anatomical description of the tumour is thus briefly given.

"1. The osteoids are irregularly knobbed (höckerige) tumours, which develop themselves sometimes slowly in the course of some years, and sometimes rapidly, growing, for the most part, first on bones and from their surface, attaining often to an enormous size, and consisting chiefly of osseous substance in the interspaces of which there is an unossified formative part, of the firmness of fibro-cartilage, such as also usually covers the surface of the osseous structure. 2. The condition of this osseous substance is sometimes very porous, and brittle, and split up on the surface into heaps of innumerable lamellæ and fibres; sometimes, on the contrary, it is firmer and more like healthy osseous tissue. The outer part of the tumour never forms a smooth rounded shell about the softer part, such as is found in enchondroma; neither is the bone ever distended into a sac. The minute structure of the osseous substance is like that of all bones. 3. The unossified part of the tumour is a grayish-white and usually firm substance, traversed by vessels knobbed on the surface, which will not tear, and has no similarity with the substance of medullary fungus. Under the microscope it appears as an indistinctly fibrous, dense network, with very small interspaces and here and there scattered primitive cells, or nuclei as remains of cells. It is in every respect similar to the animal basis of the already ossified part, and thus is prepared for ossification. It is distinguished from cartilage as well by its structure as by its chemical condition. On boiling it yields gelatine, and no chondrine. 4. These tumours result from a tendency to a morbid increasing and destructive formation of bone, which usually falls at first on one bone, but afterwards extends over other parts of the osseous system, and (which is of essential importance) attacks also other parts besides bones, so that before or after the amputation of the affected limb, osteoids completely independent of the bones of the skeleton may form in the soft parts, for instance, in the cellular tissue, the serous sacs, the lungs, lymphatic glands, or the interior of the large vessels. 5. The consecutive osteoids may appear in the looser, porous, lamellar form, as well as in that of the most

* Augustin, De Spina ventosa ossium, Halæ, 1797; Tab. iv.—J. P. Weidmann, Annotatio de Steatomatibus, Mazuntiæ, 1817; Tab. v.— Howship, Medico-Chirurgical Transactions, vol. viii, Tab. iii, figs. 1 and 2.—Haenel, Diss. de Spina ventosa, Lips. 1823. —Cooper and Travers, Surgical Essays Tab. ix, figs. 5, 6.—Cruveilhier, Anatomie pathologique, livr. 34, pl. iv.— Cheselden, Osteography, Tab. 42, pl. ii.—Sandifort, Museum anatomicum, 184, vol. iv, Tab. 54.

compact and hardest bone; so that sometimes very firm osteoids of other parts follow primary looser osteoids on the bones, or, in one and the same body some parts develop the loosest, others the firmest osteoids." (pp. 403-4.)

In support of this description, nine cases of the disease, more or less accurately observed both before and after death, are detailed; of which three were hitherto unpublished, and the six others are taken from the works cited below.* References are also given to some other cases, probably but not certainly to be proved, of the same nature. But of these cases we shall make no abstract; for they are not related in a practical or diagnostic view, the symptoms observed are not materially different from those of other malignant diseases of bone, and the results of the examinations after death are embodied in the extract already made. Taken together they have suggested to the author these following remarks on the nature of the disease.

These tumours, he believes, devolop themselves in the first instance in and from the periosteum. In some cases the limit between the new growth and the bone is distinct; and in one, at the margin of the tumour, the periosteum could be traced from the adjacent bone going over it, and then loosened and split up in bundles, which were permeated by the new substance. Besides, like periosteum, the unossified part of the tumour yields gelatine, and not chondrine, as that of the cartilaginous tumour does. But although the tumour may thus, at least usually, commence at the periosteum, it does not in its growth limit itself to it or to similar fibrous tissues, but may penetrate among the adjacent muscles, wasting them, destroying their tissue and even assimilating it. The bones themselves also may in the progress of the disease be materially affected: but herein Müller's account is very imperfect.

The production of these tumours presupposes a certain general disposition to a bone-forming vegetative irritation (!); developed in some cases in consequence of external injury, and capable, "by transplantation of the conditions of the particles of the tissue," of passing from the bone first affected to others and, at last, to soft parts. Thus the whole nutritive functions suffer and the patient if not destroyed by the local effects of the tumour dies exhausted.

In their general character these growths are so far like cancers, that Müller suggests the question whether they might not be regarded as a form of cancer and called *carcinoma osteoides.* Only, in two circumstances they differ, namely, that they rarely if ever soften, and that "the exchange of osteoids as equivalents with other cancers," (osteoids appearing after removal of carcinoma of the breast, or carcinoma following the removal of osteöid tumours,) is not yet sufficiently established. A case is related, however, in which there was a combination of the formation of bone and of soft cancer, numerous cystic medullary tumours being formed with bone in various proportions within them, either in the walls of their cells or pervading their substance.

* Observatio H. Laubii, Spina ventosa femoris sinistri, vomicæ pulmonum duræ. Acta med. phys. nat. cur. vol. i, 1727, p. 318.—Cheston, in Philosophical Transactions, 1780, vol. 70.—Catalogue of the Hunterian Museum, 1830, part i, Nos. 512, 532, 533, and part ii, Nos. 461, 462; and Baillie's Morbid Anatomy, as already quoted.—Ph. v. Walther, Ueber Verhartung, Skirrhus, Krebs, &c. in his Journal, B. v, p. 290.—Catalogue of the Museum of St. Bartholomew's Hospital, Ser. i, No. 108.—Philosophical Transactions 1740, p. 616.

In a kind of appendix to his Paper, Müller endeavours to give some account of what were really the diseases which writers have included under the vague, general expressions "spina ventosa," "osteosteatoma," &c. and then, after no brilliant success in this attempt, points out the diagnosis (post-mortem) between the osteoid tumour and those with which it may be confounded, namely, the enchondroma or cartilaginous tumour, non-malignant osteo-sarcoma, osteo-phytes, fibrous or dermoid tumour, medullary tumour of bone, and the osteo-cystoid or cystic tumour of bone. But on this part, although some few good observations are scattered, there is nothing tangible enough for our purpose, except the following interesting account of a case of "telangiectasie," erectile tumour, or aneurism by anastomosis, in which the disease assumed some of the characters of the malignant tumours.

"The same relation which we observe between the innocent sarcoma and the medullary sarcoma, and between the innocent, often ossifying desmoid tumour and the osteoid, may be observed between the innocent telangiectasis and a cancerous incurable form of this disease. It is well known that telangiectasis is, in general, an innocent disease. But a case has occurred to me which presented the complete structure of telangiectasis with the distinct malignant character, and has induced me, with some other extant analogous observations to consider a carcinoma telangiectodes s. cirsoides, as well founded. The case is that of a woman of cachectic habit, whose arm was amputated in consequence of a deep-seated tumour between the muscles and in the muscles themselves of the forearm, which were softened and in parts infiltrated by a yellowish brown structureless substance. The tumour consisted almost entirely of very considerable dilatations of softened blood-vessels and extravasations of blood. . . . . . . . . Half a year afterwards Dr. Reich brought me some new formations which had been found in the examination after death of the same person, in her abdomen. They were large masses consisting of branches of dilatations of blood-vessels as large as crow-quills from the omentum, and similar degenerations from some other organs of the abdomen; and all were completely filled with blood. . . . . The case is a parallel of one published by P. H. v. Walther.* In this, a young man who had two nævi on the leg which were removed in consequence of their increase, died two years and a half afterwards of sudden and uncontrollable hæmoptysis; and after death, knots were found in his lungs which consisted for the most part of dilated blood-vessels."

Two probably similar cases are referred to: one by Froriep in the 'Encyclopädisches Wörterbuch,' Bd. xiii, in which there were numerous tumours in the uterus, consisting chiefly of dilated blood-vessels, with proportionally small quantities of medullary substance, and in the lungs numerous reddish brown knots. The other case is that delineated by Cruveilhier in the twenty-third Livraison of his 'Anatomie Pathologique,' as an instance of numerous erectile tumours. But when these cases are quoted as examples of erectile tumours or telangiectasis multiplying themselves after the fashion of cancers, we must ask for further evidence, because many tumours (e. g. fatty and cartilaginous tumours) are produced in considerable number in the same person without having any other character in common with the malignant class, and because it is not proved that in the first two cases the tumours found in internal organs after death had not existed in them as nævi from birth. Such nævi are not very uncommon; nor would their presence be indicated, perhaps, till they began to enlarge as other nævi do, one knows not wherefore.

* Graefe u. Walther's Journal, Bd. v, p. 261, and V. Walther's Abhandlung über Verharting, Skirrhus, &c. Beob. xv.

